# Analysis of Endocytic Pathways in *Drosophila* Cells Reveals a Conserved Role for GBF1 in Internalization via GEECs

**DOI:** 10.1371/journal.pone.0006768

**Published:** 2009-08-26

**Authors:** Gagan D. Gupta, Swetha M. G., Sudha Kumari, Ramya Lakshminarayan, Gautam Dey, Satyajit Mayor

**Affiliations:** National Centre for Biological Sciences, Bangalore, India; University of Geveva, Switzerland

## Abstract

In mammalian cells, endocytosis of the fluid phase and glycosylphosphatidylinositol-anchored proteins (GPI-APs) forms GEECs (GPI-AP enriched early endosomal compartments) via an Arf1- and Cdc42-mediated, dynamin independent mechanism. Here we use four different fluorescently labeled probes and several markers in combination with quantitative kinetic assays, RNA interference and high resolution imaging to delineate major endocytic routes in *Drosophila* cultured cells. We find that the hallmarks of the pinocytic GEEC pathway are conserved in *Drosophila* and identify *garz*, the fly ortholog of the GTP exchange factor GBF1, as a novel component of this pathway. Live confocal and TIRF imaging reveals that a fraction of GBF1 GFP dynamically associates with ABD RFP (a sensor for activated Arf1 present on nascent pinosomes). Correspondingly, a GTP exchange mutant of GBF1 has altered ABD RFP localization in the evanescent field and is impaired in fluid phase uptake. Furthermore, GBF1 activation is required for the GEEC pathway even in the presence of Brefeldin A, implying that, like Arf1, it has a role in endocytosis that is separable from its role in secretion.

## Introduction

Internalization of cargo at the cell surface takes place via multiple mechanisms. For instance, the well-studied transferrin receptor (TfR) is endocytosed via the dynamin-mediated pinching of clathrin coated vesicles from the plasma membrane [1], [2]. Other pathways that do not require clathrin or dynamin for internalization are less well characterized, but are also prevalent - the GEEC pathway is one such example [3]. This pathway was originally identified on the basis of the selective internalization of various GPI-APs such as the folate receptor [4], but has also been shown to facilitate the entry of: cholera toxin bound to its ganglioside receptor GM1[5]; the vacuolating toxins aerolysin and VacA[6]; and bulk fluid phase (pinocytosis) in diverse mammalian cells [4].

At the cell surface, a fraction of GPI-APs are organized in nanoclusters which are sensitive to the extraction of cholesterol [7]. The cholesterol-dependent organization of GPI-APs, as well as their endocytosis via the GEEC pathway, requires cortical actin polymerization [8], [9]. Recent evidence links local actin polymerization to GEEC endosome formation via a regulatory circuit of two small GTPases - Cdc42 and Arf1[8], [10]. Here, Cdc42 cycles on or off the plasma membrane according to its active (GTP-bound) or inactive (GDP-bound) state respectively, where it promotes actin polymerization [8]. Cdc42 cycling is modulated by ARHGAP10, a Rho GTPase- activating-protein (GAP) that also harbours an Arf-binding domain which is specific for Arf-GTP (ABD; [10], [11]). Thus Cdc42–GAP activity, and actin-driven GEEC endocytosis is coupled to Arf1 activation [10]. However, it is not known how Arf1 activation occurs during GEEC endocytosis.

Arf1 activation is facilitated by guanine-nucleotide exchange factors (GEFs; [12]), which contain a conserved Sec7 domain responsible for GDP-GTP exchange [13], [14]. Two well characterized Arf-GEF subfamilies, GBF1 and BIGs, appear to regulate Arf activation for coat recruitment and vesicular traffic at the Golgi complex [15], [16]. It follows that these Arf-GEFs are predicted *in vivo* targets for the fungal metabolite Brefeldin A (BFA), an established inhibitor of the secretory pathway which binds at the interface between the Sec7 domain and Arf-GDP *in vitro* [17], [18]. Since the GEEC pathway is upregulated in the presence of BFA, it has been proposed that the effector GEF(s) required to activate Arf1 for GEEC endocytosis may be BFA-insensitive [10].

The significance of the GEEC pathway is broadening as it is identified in more mammalian cell types and additional cargo is found [3]. Furthermore, evidence from *Drosophila* cells indicates that the GEEC pathway may be conserved across phyla. In hemocytes derived from *shibire* (*Drosophila* dynamin) temperature-sensitive mutants, GPI-APs and the fluid phase continue to be internalized at the restrictive temperature [19]. However, the roles of key molecules such as Arf1, Cdc42, cholesterol and actin with respect to endocytosis are unexplored in *Drosophila*, so the presence of a canonical GEEC pathway in this organism is still an open question.

In this report, we develop quantitative endocytic assays in *Drosophila* S2R+ cultured cells using a variety of fluorescent probes, reporters and high resolution microscopy. We then exploit the facility of long double-stranded RNA (dsRNA) mediated RNA interference in this cell line (RNAi; [20]) to establish the presence of an Arf1-mediated pinocytic pathway that bears a striking resemblance to the GEEC pathway. With the added benefit of lower gene redundancy in the *Drosophila* genome [21], we utilize functional RNAi genomics to identify the Arf GEF, *garz*, as a candidate Arf1 effector during pinocytosis in S2R+ cells. We validate this strategy by examining the role of the mammalian ortholog of *garz*, GBF1, and find that it is a novel regulator of the GEEC pathway.

## Materials and Methods

### Materials

Apart from the fluorochromes Cy3, Cy5 (AP Biotech, UK), Alexa488/568/647, FITC and Latrunculin A (Molecular Probes, OR), all chemicals were obtained from Sigma-Aldrich, MO. Hybridoma cultures containing anti-GFP monoclonal (1B3A8) and anti-hTFr monoclonal (OKT9) were obtained from S. Sundaresan (Bangalore Genei, India) and American Type Culture Collection (ATCC,VA) respectively, and purified with IgG Fast Flow columns (Amersham,UK). Probes were labeled with Alexa or Cy3/Cy5 fluorophores according to manufacturers' instructions. Anti-*Drosophila* dynamin antibodies were from BD Biosciences, CA. Secondary antibodies were from Jackson Laboratories, ME. PI-PLC was purified from PI-PLC-expressing bacterial strains [22].

### Plasmids and Constructs

pUAST hTFr encoding the human transferrin (Tf) receptor (TfR), and pCasper YFP-Rab7 [23] were kind gifts from S.Cohen (EMBL, Heidelberg) and S.Eaton (MPI Cell Biology, Dresden) respectively. pBSPURO was obtained from M. Wilm (EMBL, Heidelberg). pUAST-GFPRab5 and pUAST-GFPRab11 were cloned by ligation of PCR generated fragments of GFP and *Drosophila* Rab5 or Rab11 cDNAs into the pUAST vector [24], and subsequently sequenced. pUAST-GFPGPI [25] was obtained from the *Drosophila* Genome Resource Center (DGRC, IN). Mammalian GFPGBF1 and GFPGBF1^E794K^ constructs were a kind gift from E. Sztul (U. Alabama, Birmingham).

### Cell culture, Fly stocks, RNAi and stable lines

*Drosophila* S2R+ cells were obtained from S. Yanagawa [26], split every 6–8 days and grown at room temperature (21–24°C). They were maintained in Schneider's *Drosophila* Insect Medium (SDM; Invitrogen) supplemented with 7.5% heat-inactivated fetal bovine serum (FBS; Gibco-BRL) and 150 µg/ml penicillin, 250 µg/ml streptomycin, and 750 µg/ml glutamine. RNAi was performed according to the procedure of Clemens [20]. Transfections were performed with Cellfectin (Invitrogen, CA) or Effectene (Qiagen, Germany), according to the manufacturer's instructions. For the generation of stable lines, the PBSPURO plasmid bearing puromycin N-acetyltransferase was co-transfected at a 1∶30 ratio with the desired constructs. Stable lines were selected over 2–3 weeks by incubation in medium containing 1 ug/ml puromycin. Chinese Hamster Ovary (CHO) cells stably expressing FR-GPI and human TfR (IA2.2 cells) were used for endocytic assays. CHO cells were grown in HF-12 (HiMEDIA, Mumbai, India) containing NaHCO3 and penicillin, streptomycin (100 µg/ml) and supplemented with 10% FBS (GibcoBRL, Rockville, MD). CHOs were transfected with different DNA constructs using FuGENE6 (Roche Diagnostics, Germany) according to standard protocol, and assayed 18–20 hrs after transfection [10].

### Probes for endocytosis and immunodetection

Since both the S2 line and primary cultures of *Drosophila* hemocytes have been previously shown to exhibit scavenger receptor-endocytosis [19], [27], we tested for the presence of this pathway in S2R+ cells using a polyanionic ligand (malelyated BSA; mBSA; [19], [28]). The 10 kDa fluid phase tracer FITC-dextran was made as described previously (Fdex; [4]) and 10 kDa tetramethyl rhodamine-dextran (Rdex) was purchased from Molecular Probes, OR. Human apo-transferrin was iron-loaded and conjugated to Alexa 568 or Alexa 647 dyes [29], [30]. Anti-GFP hybridoma was purified (see Materials) and conjugated to Alexa 647 to yield A647αGFP. Immunofluorescence was carried out as described [31]. For Tf and GPI-AP probes, stable S2R+ lines constitutively expressing the well characterized human transferrin (Tf) receptor (TfR) and/or GFP-GPI were generated. Cy3 conjugated mBSA (Cy3mBSA; [19]) or Alexa568/647 conjugated Tf (A568Tf/A647Tf) was used to probe receptor-mediated pathways whereas FITC or TMR conjugated to Dextran (FDex and RDex respectively) were used as fluid-phase tracers. The receptor probes Cy3mBSA, Cy5mBSA and biotinylated Cy3mBSA (Cy3bmBSA; [19]) were used at 10–30 µg/ml in S2R+ cells. At these concentrations, the binding and internalization of labeled mBSA was completely competed by 100 fold excess unlabeled mBSA. Fdex and Rdex, which are markers for bulk fluid uptake, were used at 1–2 mg/ml, unless otherwise specified. Tf and αGFP probes were used at 10 ug/ml and 5 ug/ml respectively. In the corresponding cells, Cy3mBSA, A568/A647 Tf and αGFP ligands show saturable receptor labeling, which is competed by 100 fold excess unconjugated ligand (not shown).

### Uptake assays

For S2R+ uptake experiments, cells were incubated with endocytic probes in M1 buffer (150 mM NaCl, 5 mM KCl, 1 mM CaCl_2_, 1 mM MgCl_2_, 20 mM HEPES, pH 6.9; modified from [29]) supplemented with BSA (1.5 mg/ml) and D-glucose (2 mg/ml) at room temperature (21–24°C), unless otherwise indicated, and extensively washed in the same medium. To visualize the specific endocytic uptake of GFP-GPI or TfR, cells were incubated with fluorescent conjugates of αGFP or Tf (along with fluid phase markers if needed) at room temperature for different pulse times and then washed in M1 and placed on ice. Where necessary, PI-PLC (0.3 mg/ml; 45 min on ice) or ascorbate buffer with 50 ug/ml desferroxamine mesylate (pH 4.5; 10 min on ice) treatment was carried out to remove cell-surface GFP-GPI or Tf respectively. Surface pools of receptors were labeled by further incubation on ice with antibodies to GFP (αGFP with a different fluorochrome) or anti-hTfR (Okt9) for 30 min. Subsequently, cells were washed, fixed (2.5% paraformaldehyde in M1) and imaged. When required, endosomal pH was neutralized by the addition of ammonium chloride (30 mM in M1) post-fixation so as to dequench pH-sensitive fluorochromes. Assays for surface accessibility of mBSA were carried out as described previously [19]. Mammalian uptake experiments were carried out exactly as described previously [10]. For Brefeldin A (BFA) experiments, IA2.2 cells were treated with 20 µg/ml BFA for 60 min and pulsed with TMR-Dex during the last 10 min before fixation and imaging.

For determining externalization rates in S2R+ lines (see [32]), cells were incubated in the presence of a saturating concentration of A568 Tf for varying times until they achieved a maximum, steady-state value of cell-associated label. Since Tf binding to surface TfR is rapid, and since saturating levels of Tf were used in this assay, the rate of accumulation of Tf is mainly dependent upon the rate at which unoccupied intracellular TfR are externalized to the plasma membrane to acquire Tf. The exocytic rate constant, k_e_ can be quantitated once the parameters Tf_s_ and Tf_ss_ are estimated from a best fit to the function:
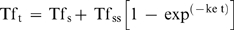
where Tf_t_ is the total amount of Tf bound to TfR at time t; Tf_s_ is the amount of Tf bound to surface TfR (constant in this assay); and Tf_ss_ is the Tf bound to internal TfR at steady state. The values for k_e_ are determined from the best least-squares fit to this function [29]. As a control, to evaluate the contribution of the biosynthetic pool of TfR during the course of the assay, cells were pretreated with 75 µM cyclohexaminde for 2 hrs and the assay was carried out in the presence of cyclohexamide (Figure S2B).

### Cholesterol measurements and serum delipidation

FBS was delipidated by organic extraction with diethyl ether according to Cham and Knowles [33], and dialysed extensively in PBS. This extraction reduced total cholesterol content by over 99% as measured by the Red Amplex Cholesterol Assay Kit (<10 ug/ml sterol; Molecular Probes, OR), while leaving overall protein levels in serum unchanged. S2R+ cells were adapted to growth in delipidated medium containing 10% lipid-extracted FBS in SDM. This was achieved by serial passage in 150 ml flasks with linearly increasing levels of lipid-extracted FBS over 5–8 weeks. Filipin staining and methl-β-cyclodextin (MβCD) treatments were as described previously [8] except that the incubation temperature was reduced to 22°C to match optimum growth conditions for *Drosophila* cells.

### Fluorescence imaging, quantification and processing

Wide field imaging was carried out on a Nikon TE300 inverted fluorescence microscope and images captured with a CASCADE II camera (Photometrics, AZ). Fluorescence quantification was carried out with a low magnification objective (20x, 0.75NA) to obtain 30–100 cells per field, while higher resolution images for visualization purposes were obtained with a 60X, 1.4NA objective. Confocal microscopy was carried out on an Andor Spinning Disc confocal imaging system (60x 1.42 NA or 100x 1.4 NA, with Ixon EMCCD; Andor Technologies, Ireland) using appropriate factory-set filters and dichroics for different fluorophores. Care was taken to avoid fluorophore saturation during acquisition. Total internal reflection fluorescence (TIRF) microscopy imaging was done on a custom-built setup around a Nikon Eclipse TE 2000U inverted microscope (described in detail in [8]). Images were collected using appropriate filters onto cooled EMCCD-based Cascade 512B cameras (Roper Scientific, AZ) with ‘on-chip multiplication gain’ feature for providing single-molecule detection sensitivity in solutions and cells. For two-colour TIRF microscopy, cells were illuminated sequentially by 488 or 543 nm lasers and fluorescence emission was collected using corresponding emission filters mounted on a shutter wheel (Sutter Instruments, CA) operating coordinately with the illuminating lasers. Background subtraction and fluorescence quantitation was carried out as described previously [29] using MetaMorph or custom routines written in MATLAB (described in Figure S1). Statistical methods were as described previously [10]. Images were pseudo-coloured using Adobe Photoshop and composites were assembled using the same software. Quantification of colocalization was performed as described [4], [34]. All processing including determination of colocalization was performed using similar parameters regardless of the type of endocytic tracer used. Colocalization fraction was calculated as the number of endosomes per cell whose surface area overlapped at least 30% in each cell. The maximum extent of colocalization obtained by this method is 80% for cointernalized A647-TfR and A568-TfR in the same cell [4].

### RT-PCR and Western blotting

For each condition or dsRNA treatment, about 10 ug total RNA was extracted from ∼10^6^ S2R+ cells with Trizol reagent (Sigma). First strand cDNA synthesis was performed with Superscript III Reverse Transcriptase (Invitrogen, CA) and a polyT22 primer according to the manufacturer's instructions. The reaction product was then treated with DNAse-free RNAse (Ambion, CA) to remove RNA template. Gene-specific primers spanning introns were designed to confirm the absence of genomic DNA contamination during PCR for each transcript. PCR was performed for 20 cycles and 30 cycles for semi-quantitative comparison of amplification. Western blotting was performed according to standard protocols.

## Results

### Pathways of endocytosis in *Drosophila* S2R^+^cells

**Figure 1:**
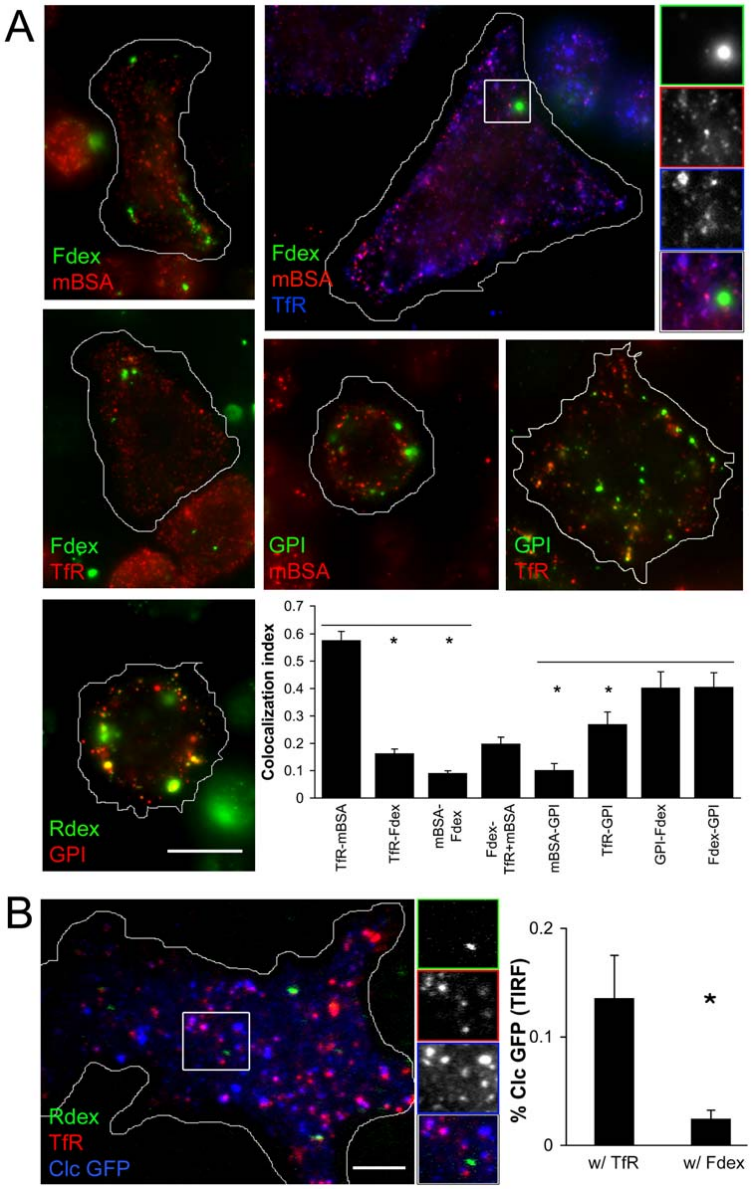
Fluid phase and GFP GPI probes mark a distinct endocytic route at early time points in S2R+ cells. (A) FITC- or TMR- dextran (Fdex, Rdex respectively) was pulsed along with: malelyated BSA or A568/A647 TfR (mBSA, TfR respectively); or αGFP (to mark internalized GFP GPI) for 30–60 s. Cells were fixed and imaged with wide field microscope at high resolution. Colocalization of different probes in combination with each other was then quantified. Bar graph represents mean±s.e.m. from at least 3 independent experiments with 20–50 cells for each probe combination in each experiment. (B) Rdex was co-pulsed with A647Tf for 30 s in cells expressing Clathrin light chain GFP (Clc GFP) which were fixed and imaged with TIRF microscopy. Bar graph represents the mean±s.e.m. percentage of Clc GFP spots that colocalized with TfR and Rdex endosomes (measured from >600 endsomes of each type from 13 cells). Asterix on all bar graphs mark the comparison population mean value and refer to those means that are significantly different from controls (P<0.05 by Student's t-test). The outline in white was traced using the phase contrast image of the cells obtained in bright field. Bars in A = 5 µm, B = 2 µm, insets magnified 2x.

The S2R+ cell line, which expresses a *wingless* pathway receptor [26] is believed to be a derivative of the embryonic S2 line [35]. The morphology of these cells is heterogeneous, but the majority of cells spread out, exhibiting broad and thin lamellapodia [36]. These characteristics allow for high resolution wide field imaging of early endosomes. To visualize early steps of internalization, we co-pulsed S2R+ cells with fluid phase (Fdex/Rdex) and receptor (Cy3mBSA and/or A647Tf) probes for very short times (30–60 s) and fixed them before imaging (see Materials and Methods for details of probes). Post-pulse most of the Fdex and Cy3mBSA probes were found in separate, peripheral structures (Figure 1A). By contrast, Cy3mBSA was largely found together with internalized Cy5Tf in the same period (Figure 1A). Quantification of colocalization of these structures indicated that Tf puncta were ∼5 fold enriched in mBSA when compared to Fdex (graph in Figure 1A). Conversely, only a small fraction of Fdex punctae also contained Tf and mBSA when all three probes were co-pulsed (Figure 1A).

To follow the endocytic route of GPI- anchored proteins (GPI-APs), S2R+ cells stably expressing GFP-GPI were pulsed with Alexa 647-coupled antibodies to GFP (A647αGFP) and Rdex (Figure 1A). Internalized Rdex labeled structures contained significantly more GFP-GPI as marked by A647αGFP, compared to mBSA or Tf (graph in Figure 1A). To examine nascent endosomes present close to the plasma membrane at higher resolution, we pulsed cells expressing clathrin light chain GFP with Rdex and A647Tf for 40 s and imaged them with TIRF microscopy. In the evanescent field, the fraction of clathrin GFP punctae that colocalized with Tf at early times was over 10 fold greater than those that contained Rdex (Figure 1B).

### Sequence of endosome mixing and separation, and the fate of endocytic cargo

**Figure 2:**
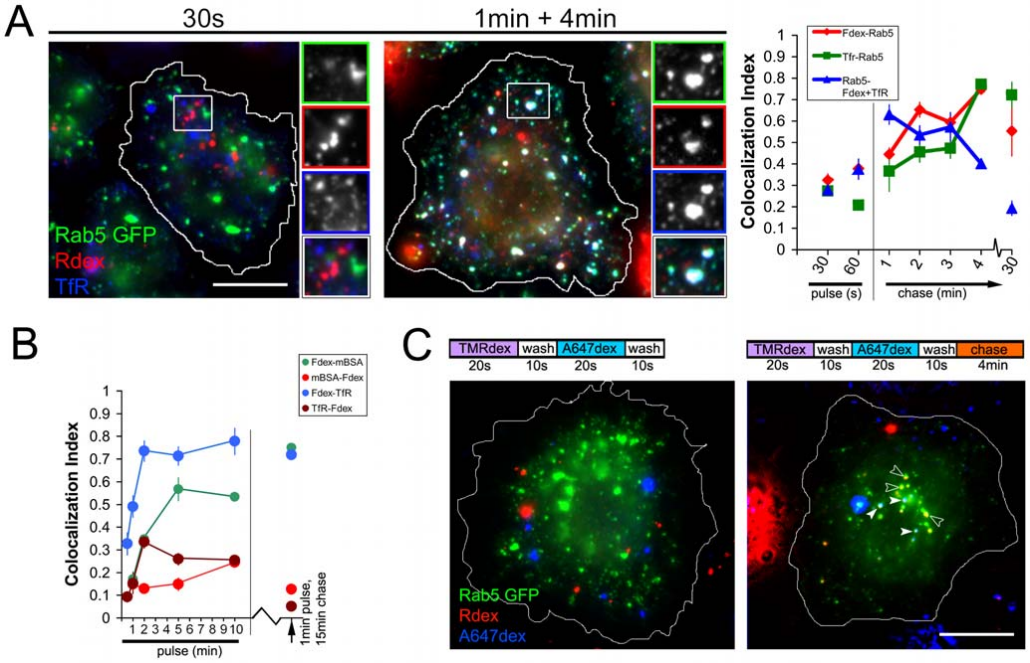
Fluid phase and TfR pathways intersect in Rab5-positive compartments and subsequently follow expected fates. (A) TMR-dextran (Rdex) was co-pulsed with A647Tf (TfR) in cells expressing Rab5 GFP (Rab5 GFP) for 30 s (left pane) or the pulse was chased for 4 min in complete medium (right pane). Graph shows quantified colocalization indices over different pulse (30 s or 60 s) and pulse and chase times (1 min pulse and 1, 2, 3 or 4 min chase; and a 3 min pulse and 30 min chase) of: Tf-labeled endosomes co-localized with Rab5 GFP (blue symbols and line); Rdex endosomes with Rab5 GFP (red symbols and line); and Rab5 GFP-labelled endosomes that contained both Rdex and Tf (green symbols and line). Data points represent mean±s.e.m pooled from 2 independent experiments with at least 20 cells for each point. (B) Time course of mixing of Fdex, mBSA and TfR probes in S2R+ cells. Colocalization values for different probes and pulse times (0.5 min, 1 min, 2 min, 5 min and 10 min; or a 15 min chase following a 1 min pulse) were obtained as described earlier (Figure 1A). The fraction of TfR or mBSA endosomes that also contain Fdex is relatively low at all time points [TfR-Fdex (brown symbols and line) and mBSA-Fdex (red symbols and line), respectively]). Conversely, the fraction of Fdex endosomes that contain TfR (Fdex-TfR; blue symbols and line) or mBSA (Fdex-mBSA; green symbols and line) is initially low, and increases with longer pulse times. (C) S2R+ cells expressing Rab5 GFP were pulsed with Rdex, washed and then pulsed with A647dex to examine homotypic fusion of newly formed fluid endosomes (see schema at the top of image). Rab5 GFP was not found on endosomes labeled with either the first or the second short pulse of dextran (left panel). When the two staggered pulses were chased for 4 min (right panel), endosomes containing the first pulse acquired Rab5 GFP (empty arrowheads) and a fraction of these also contained the second pulse (filled arrowheads). There was little or no mixing between the two pulses outside Rab5 GFP labeled structures. Bars in A, C = 5 µm, insets magnified 2x.

To study endosomal dynamics, we generated a stable S2R+ line expressing moderate levels of Rab5 GFP, an early endosomal marker which participates in homo- and hetero-typic endosome fusion and sorting steps [37], and performed pulse-chase assays with Rdex and A647Tf. When levels of colocalization with the steady state pool of Rab5 GFP were examined over different chase times, most Rdex and A647Tf containing endosomes were found to lack Rab5 during the short pulse (30 s–60 s), and then acquire Rab5 steadily over a chase time of upto 30 min (Figure 2A). Interestingly, mixed endosomes overlapped with Rab5 punctae in a biphasic pattern, rising rapidly to encompass over 60% of Rab5 punctae at 2 min post probe entry and falling to pre-pulse levels with longer chase times (Figure 2A). This time course is not influenced by overexpression of Rab5-GFP protein, as mixing kinetics of Fdex and Tf in lines with or without Rab5 GFP was similar (Figure S2A). Furthermore, heterotypic mixing between Fdex and TfR probes in wild type cells also rises rapidly to a peak in a 2 min pulse (Figure 2B), a process that in mammalian cells has been shown to require Rab5 function [38]. A similar time course of mixing was found when examining homotypic fusion between fluid-containing endosomes, which always occurred in Rab5 marked structures (Figure 2C).

**Figure 3:**
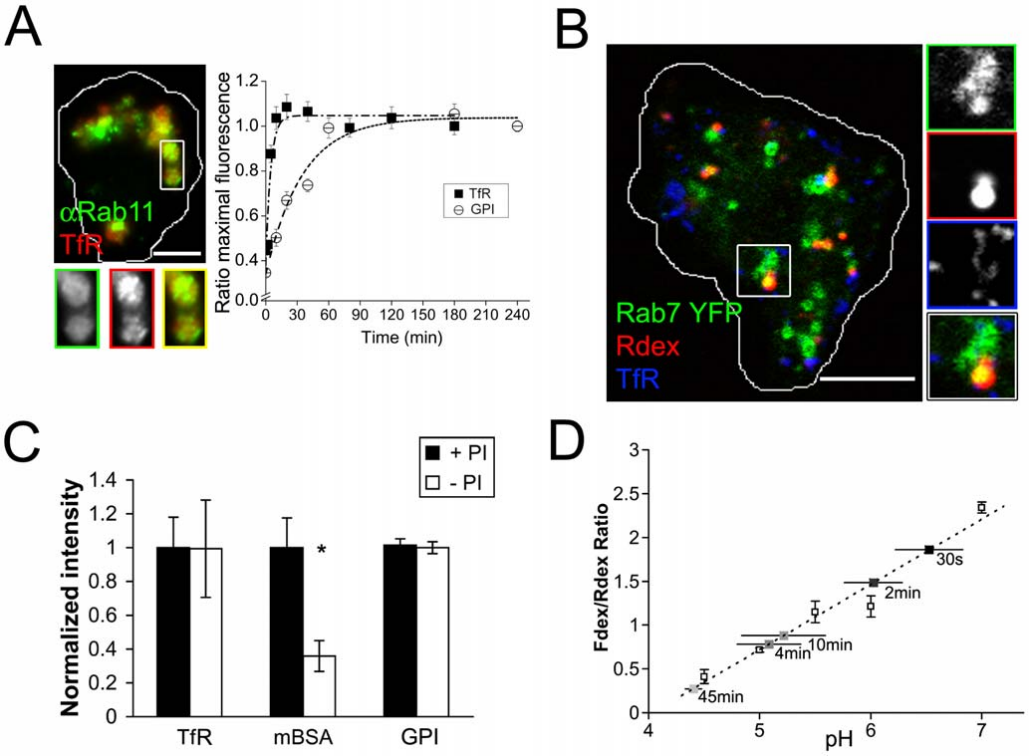
Characteristics of endosomal progression in S2R+ cells. (A) A568Tf (TfR) was pulsed for 15 min and cells were processed for immunocytochemistry with anti-Rab11 (αRab11), and subsequently imaged at high resolution. Plot shows kinetics of Tf and αGFP uptake in S2R+ cells transfected with the either the human TfR or GFP-GPI in an approach to steady state assay (see Materials and Methods). Each data point represents the mean±s.e.m. of a replicate experiment from at least 200 cells/replicate. Points have been fit to y = 0.381+0.666(1 – e^-0.216t^), R^2^ = 0.922 for TfR and y = 0.339+0.698(1 - e^-0.029t^), R^2^ = 0.966 for GFP GPI. (B) TMR-dextran was co-pulsed with A647Tf for 15 min in cells expressing Rab7 YFP, and cells were imaged with a spinning disk confocal. A single (median) plane from a stack is shown. (C) Cells expressing corresponding receptors were pulsed with TfR, mBSA or αGFP for 2 hrs and then the probes were chased in complete medium for an additional 2 hrs in the presence (+PI) or absence (-PI) of protease inhibitors. The integrated intensity of each probe in cells was then quantified and normalized to controls. Values are represented as mean±s.e.m. from at least 100 cells per treatment in 2 independent experiments. Asterix denote P<0.05 by Student's t-test. (D) Endosomal pH measurements. S2R+ cells were pulsed for 30 min with Fdex and Rdex, washed and mildly fixed, and subsequently equilibriated with a range of different buffer pHs to obtain a linear calibration of the ratio of Fdex/Rdex fluorescence against pH (dotted line through empty circles; R^2^>0.95). To label different populations of endosomes, S2R+ cells were either pulsed for 30 s with Fdex and Rdex or pulsed and then chased for 2 min, 4 min, 10 min and 45 min (shown on the graph as squares filled in descending grayscale). The ratio of Fdex/Rdex intensities was measured and compared to equilibriated values to obtain pH values. 30 s fluid endosomes were mildly acidic with a pH of 6.5±0.3 (mean±s.e.m. of 2 independent experiments, >30 cells for each time point) whereas the pH of more mature fluid endosomes was progressively more acidic, with 45 min endosomes having a pH of 4.4±0.1, close to values reported for lysosomes. Bars in A = 2.5 µm, B = 5 µm, insets magnified 2x.

We next examined the kinetics of Tf binding and recycling in *Drosophila* cells in detail because it is unclear whether the canonical Tf pathway operates in insect lines [39]. The binding and uptake of TfR was kinetically analyzed over 2 hrs with saturating levels of A568Tf (Figure 3A) in an approach to steady-state assay (Materials and Methods; [32]). At each time point, total binding of Tf at room temperature was normalized to levels of Tf bound out to steady state (upto 2 hrs). Total binding at 4°C was assumed to be the initial surface receptor occupancy. Uptake at room temperature exceeded binding at 4°C by 2.5-fold and saturated after 15–20 min, and similar values were obtained when the assay was carried out with cyclohexamide (to block synthesis of new TfR; Figure S2B). The kinetics of approach to steady state reflect the rate of export of the TfR to the cell surface with a rate constant of 0.216/min (t_1/2_∼3 min) if we assume a single first order process. This value agrees with the time course of quantitative TfR uptake in S2R+ cells after surface receptor normalization with Okt9 (not shown). Also, this externalization rate is 3 fold higher than in CHO cells (0.069/min, t_1/2_∼10 min; Johnson et al., 1993), and ∼10 fold higher than that of GFP-GPI in S2R+ cells (0.02883/min, t_1/2_∼25 min; Figure 3A). Consistent with a recycling step, a significant pool of internalized TfR after a 20 min pulse was found in large endosomes that also labeled with antibodies to the recycling marker Rab11 (Figure 3A) and were devoid of the lysosomally directed fluid phase (Figure 3B), and unlikely to enter a degradative pathway. Consistent with this, Tf levels inside cells were not sensitive to the addition of protease inhibitors (PI; Figure 3C).

After a 20 min pulse, the majority of internalized endosomes that accumulated Fdex were marked by YFP-Rab7 (Figure 3B), and co-localized with Cy3-mBSA (not shown; [19]). Internalized Cy3mBSA, a marker that traverses the lysosomal route (Sriram et al., 2003), is protected from degradation in the presence of PI (Figure 3C). We also monitored the change in pH accessed by fluid-phase markers, upon increasing chase time in the endocytic pathway. Consistent with Rab5 acquisition in sorting endosomes, the pH of fluid-containing endosomes 2 min after internalization, as measured by a ratiometric assay[38], is ∼6.0 (Figure 3D; [40]). This value drops to pH 4.5 once the probes reach deeper in the endocytic pathway (Figure 3D). Thus, S2R+ cells also have an endocytic system that resembles that of a typical mammalian cell (early endosomal [41]; late endosomal transport [42]). Here, endocytic trafficking involves a hetero and homo-typic mixing of cargo at the early endosomal compartment, followed by a segregation of certain membrane cargo (TfR and GPI-APs) towards a recycling fate, and lysosomal direction of the fluid-phase and ligands of the scavenger receptor.

### A dynamin- independent pinocytic pathway in S2R+ cells

**Figure 4:**
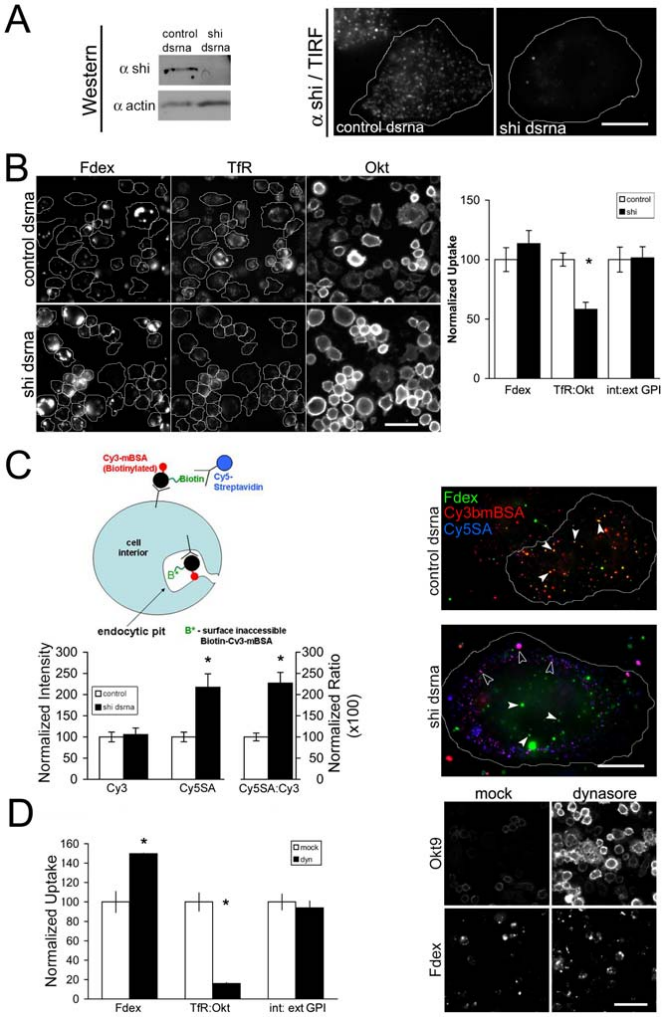
A dynamin insensitive endocytic machinery operates in S2R+ cells. (A) Specific depletion of *Drosophila* dynamin by dsRNA against the *shibire* (shi) gene as shown by Western detection of cell homogenates after 4 days depletion (left panel). Cells were also processed for immunocytochemistry followed by TIRF imaging of dynamin puncta close to the plasma membrane (right panel). (B) Cell populations were depleted of dynamin or control (zeocin) dsRNA for 4 days, and uptake of Fdex, Tf as well as surface TfR levels (Okt) were quantitated. Representative cells (outlined) from each dsRNA population are shown in all three channels and the bar graph (right panel) shows population means±s.e.m. of 2 independent experiments with >200 cells in each condition. A quantitation of internalized GFP-GPI under the same conditions is shown alongside. (C) A quantitative surface accessibility assay [19] was employed to examine scavenger receptor availability in *shibire* (*shi*) depleted S2R+ cells. The assay measures the extent to which receptors labeled with biotinylated Cy3mBSA (Cy3bmBSA) are accessible to exogenous Cy5-Streptavidin (Cy5SA; see cartoon) after they are allowed to internalize the labeled ligand (x axis legends: Cy3 - binding capacity; Cy5SA - surface accessibility to Cy5-SA; and Cy5SA/Cy3 - relative surface accessibility). The bar graph shows that after 3 min of pulse followed by 10 min of chase, *shi* depleted cells show ∼twofold higher levels of Cy5SA binding when compared to controls (mean±s.e.m.; 2 experiments, >30 cells in all cases), although they bind the same amount of Cy3bmBSA. Furthermore, while Fdex continued to be internalized and trafficked towards the center of *shi*-depleted cells (filled arrowheads in right panel), the large majority of Cy3bmBSA labeled structures were found at the cell periphery, largely accessible to Cy5SA (empty arrowheads). By this time, in control cells, Cy3bmBSA and Fdex had mixed extensively in central structures which were inaccessible to Cy5SA (marked with filled arrowheads). (D) The bar graph shows normalized uptake of Fdex, TfR and GFP-GPI in S2R+ cells treated with 20 µM dynasore (dyn) for 20 min or carrier DMSO alone (mock). Values are mean±s.e.m. (>50 cells in all cases) from two independent experiments. Representative images in the right panel are scaled equally to show the marked difference in Okt9 levels in cells after dynasore treatment, while Fdex intensities are unchanged or higher compared to the mock treatment. Bars in A = 2.5 µm, B = 20 µm, C = 5 µm, D = 20 µm.

We next assessed if endocytic trafficking of any of the endocytosed cargo had a requirement for dynamin, one of a set of key molecular criteria used for the characterization of endocytic pathways [3]. S2R+ cells were treated with control dsRNA against the gene for zeocin resistance (which is absent in the *Drosophila* genome) or dsRNA corresponding to ∼800 bp of coding sequence for the *shibire* gene, which codes for *Drosophila* dynamin. The dsRNA for *shibire* was effective in reducing protein levels for the target gene as seen in Western blots (Figure 4A). In control cells, TIRF imaging showed that antibodies to *shibire* label discrete puncta at the plasma membrane, which are presumably clathrin coated pits [19], [43]. This localization was lost in *shibire* depleted cells (Figure 4A). Cells depleted for dynamin were pulsed with either Fdex or A647αGFP (to label GFP-GPI) and A568Tf for 3 min. The ratio of internalized αGFP or Tf to the surface levels of the respective receptors in each cell was determined quantitatively via MATLAB routines (see Figure S1 for methodology of quantitative endocytic assays). In cells without dynamin, the internalized to surface ratio of TfR was reduced ∼50% compared to control cells (Figure 4B). The difference was largely due to an accumulation of TfR on the surface, probably reflecting a decreased rate of internalization of Tf. We verified this response to dynamin depletion using the fluorescent probe for the endogenous scavenger receptor, mBSA, in an accessibility assay (Figure 4C). The assay was used previously to assess the accessibility of clathrin coated pits to receptor ligands in *Drosophila* hemocytes harbouring a temperature sensitive *shibire* allele [19]. Here both control and *shibire* depleted cells showed similar quantitative levels of biotinylated mBSA (bmBSA) labeling, but the bmBSA in the shibire-depleted cells was ∼2 fold more accessible to streptavidin (Figure 4C, indicative of surface-arrested receptor pits [19]. The effect of dynamin depletion on Tf endocytosis was similar to that of rapid dynamin inhibition with the small molecule inhibitor dynasore ([44]; Figure 4D). Notably, the levels of normalized GFP-GPI uptake were not different from controls in *shibire*-depleted (Figure 4B) or dynasore treated cells (Figure 4D). Similarly, these cells also exhibited normal or higher levels of Fdex uptake after loss of dynamin function (Figures 4B,D), and Fdex endosomes continued to accumulate in cells which contained surface-arrested bmBSA structures (Figure 4C). Thus, dynamin depletion in S2R+ cells inhibits internalization of two classes of receptors, and increases their surface accessibility, while GFP-GPI and fluid uptake is not inhibited. These data conclusively show that although TfR and scavenger receptors (for mBSA) are internalized using dynamin-dependent mechanisms, GPI-AP and fluid-phase uptake relies on mechanisms that do not utilize dynamin.

### Actin and cholesterol dependence of endocytic pathways

**Figure 5:**
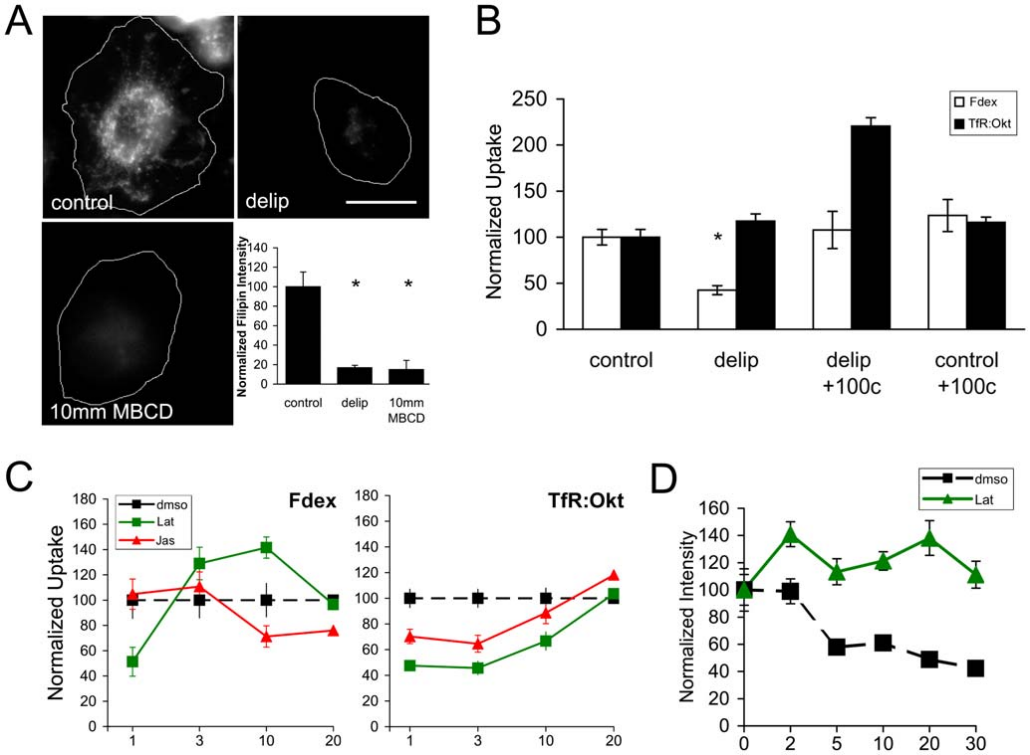
Role of actin and cholesterol in S2R+ endocytic pathways. (A) Panels shows fluorescent micrographs of filipin staining in: untreated S2R+ cells (control); cells adapted to growth in delipidated, cholesterol-free medium (delip; see Materials and Methods); and control cells treated with 10 mM MβCD for 30 min. Bar graph shows quantification of the total filipin intensity across the different treatments (mean±s.e.m., >90 cells for each treatment from 2 experiments; asterix denote P<0.05 by Student's t-test). (B) Normalized Fdex and TfR uptake in: control S2R+ cell line, cholesterol-free adapted line (delip), cholesterol-depleted lines repleted with 100 µg/ml cholesterol for 12 hrs (delip+100c) and control line grown in 100 µg/ml cholesterol for 12 hrs (control+100c). Mean±s.e.m. values are derived from 2 independent experiments with n>200 cells in each condition. Asterixs denote P<0.05 by Student's t-test. (C) Normalized uptake of Fdex and TfR in S2R+ cells over different pulse times (in minutes) in medium containing either carrier dimethyl sulphoxide (dmso); 1 µM Latrunculin A (Lat) or 4 µM Jasplakinolide (Jas). Values are represented as mean±s.e.m. at each time point/treatment, >200 cells per treatment in 2 independent experiments. (D) S2R+ cells were prepulsed for 2 hrs with Fdex and then chased in medium without probe either with Latrunculin A (Lat) or carrier alone (dmso) for increasing lengths of time in minutes. At the end of each chase time, cells were fixed and the total remaining cell-associated Fdex intensity was quantitated and normalized to control levels. Each point on the graph is represents mean±s.e.m. from 2 independent experiments with at least 100 cells each. Bar in A = 5 µm.

Since agents that manipulate cholesterol levels in cells may have pleiotropic effects and can be toxic to the cells, the precise role of cholesterol in steady state cellular processes like constitutive endocytosis has been difficult to interpret. Here we exploit the finding that *Drosophila* cells can produce lipids from metabolic pathways, but lack key genes required for cholesterol biosynthesis [45]. By substituting normal serum with delipidated serum (which contains <10 ug/ml cholesterol; see Materials and Methods) in fly growth medium, we could severely restrict the supply of cholesterol into S2R+ cells. We find that delipidated serum-adapted cells are able to grow well despite <1% of normal sterol levels in their membranes (as measured by filipin binding; Figure 5A, and Carvalho et al., submitted). Notably, these adapted cells take in significantly lower levels of Fdex in a quantitative assay (Figure 5B). Furthermore, surface levels of TfRs are reduced in these cells (not shown), but their internalization is unaffected. The specific effect of cholesterol depletion on Fdex uptake can be reversed by incubating the depleted cells in cholesterol containing medium for 12 hrs (Figure 5B). Repleted cells exhibit normal levels of Fdex uptake, and have high levels of Tf internalization when normalized to surface pools of the receptor (Figure 5B). The surface levels of TfR remain low in these repleted cells (not shown), perhaps owing to slower recovery kinetics over the 12 hr incubation period.

In S2R+ cells, both Fdex and Tf uptake were highly sensitive to the actin disrupting agent Latrunculin A (Lat) or Jasplakinolide (Jas). Cells were incubated with 1 µM Lat, or 4 µM Jas for a total of 30 min, in which time they were pulsed with Fdex or TfR in the presence of drug for different periods. Both Fdex and normalized Tf internalization was reduced >50% at short pulse times in Lat (1 min; Figure 5C). By contrast, treatment with Jas had little effect on Fdex uptake at short pulse times, while strongly inhibiting TfR uptake. At longer pulse times, Fdex accumulated in Lat treated cells, while Jas treated cells showed lower levels of Fdex uptake, and TfR uptake appeared to recover to steady state levels seen in untreated cells (Figure 5C).

To test whether the accumulation of Fdex in Lat treated cells is due to a strong inhibition of a recycling pathway that facilitates regurgitation of fluid [8], we examined cells that were pre-pulsed with Fdex and then chased over different times in the presence of Lat. Notably, a large fraction of accumulated Fdex leaves the cell over short times (<5 min) in untreated cells, while the presence of Lat inhibits this efflux (Figure 5D). In summary, our results indicate that the main features of a cholesterol and actin sensitive GPI-AP endocytic pathway that does not utilize Dynamin appear conserved in S2R+ cells.

### An Arf1- and Cdc42-dependent pathway in S2R+ cells that does not require Arf6, Rho or Rac1

**Figure 6:**
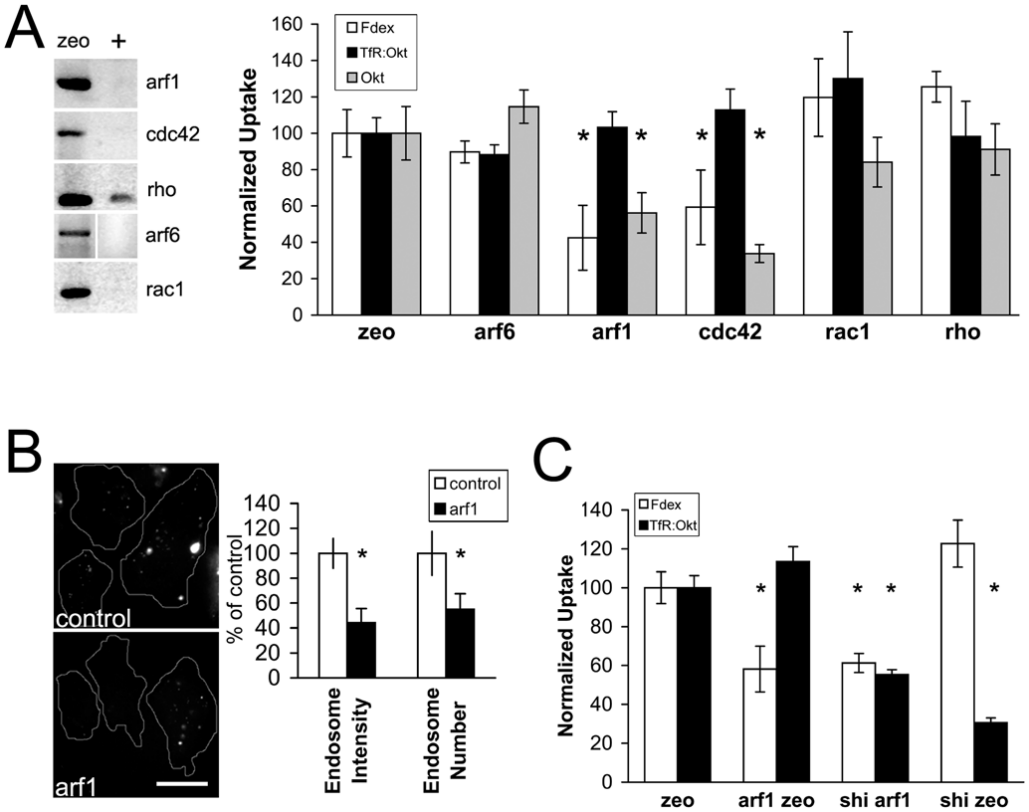
Arf1and Cdc42 but not Rho, Rac1 or Arf6 are required for dynamin independent endocytosis in S2R+ cells. (A) Depletion of mRNA of the GTPases Arf1, Cdc42, Rho, Arf6 and Rac1 as verified by reverse-transcriptase PCR on cells treated with the corresponding dsRNAs (+) or control zeocin dsRNA (zeo) for 4 days (left panel). Normalized uptake of cells depleted for these GTPases for 4 days was then quantitated as before (Figure 2B). Values are represented as mean±s.e.m. from at least 100 cells per treatment in 2 independent experiments. (B) Fdex endosomes visualized at high resolution in control and Arf1 depleted cells. Bar graph shows the average endosomal intensity and number per cell, normalized to control cells (mean±s.e.m., measured from >400 endosomes from at least 15 cells in each case). (C) Normalized uptake of cells depleted for combinations of arf1, shi and zeo dsRNAs. Mean±s.e.m. values are from one representative experiment with n>200 cells in each condition. Asterix denote P<0.05 by Student's t-test. Bar in B = 5 µm.

As a basis for a molecular understanding of dynamin independent endocytosis in S2R+ cells, we first tested the involvement of *Drosophila* orthologues of several candidate genes known from other systems (Cdc42, Rho, Rac1, Arf6 and Arf1; [3]). Depletion of the corresponding transcript after dsRNA treatment was verified using rtPCR (Figure 6A), and cells were pulsed with Fdex and A568Tf and their normalized per cell uptake was quantitated as before. As expected from their roles in the secretory pathway, Arf1 and Cdc42 were required for the surface delivery of TfR (as determined by by the reduced surface Okt9 binding compared to control cells; Figure 6A). Strikingly, uptake of Tf when normalized to the lower surface levels of Tf receptor, was not affected in cells depleted of Arf1 or Cdc42. Instead, both genes were required for optimal levels of fluid phase uptake. Rac1, Rho or Arf6 depletion did not have major effects on uptake of either probe (Figure 6A). We examined Arf1-depleted cells at higher resolution because this gene has been shown to be directly involved in the early steps of endocytosis via GEECs in mammalian cells [10]. Consistent with an early role in the process, Arf1-depleted S2R+ cells contained fewer fluid endosomes after a short pulse, and the remaining endosomes were significantly reduced in intensity (Figure 6B). To test if the pathways of uptake of TfR and Fdex were indeed distinct as dynamin- and Arf1- dependent respectively, we co-depleted cells with dsRNA against both Arf1 and *shibire*. Fdex uptake was only reduced in the presence of Arf1 dsRNA, and this inhibition was not affected by the presence or absence of *shibire* dsRNA (Figure 6C).

### Conserved role of *garz* and its mammalian counterpart GBF1 in Arf1-mediated endocytic uptake

**Figure 7:**
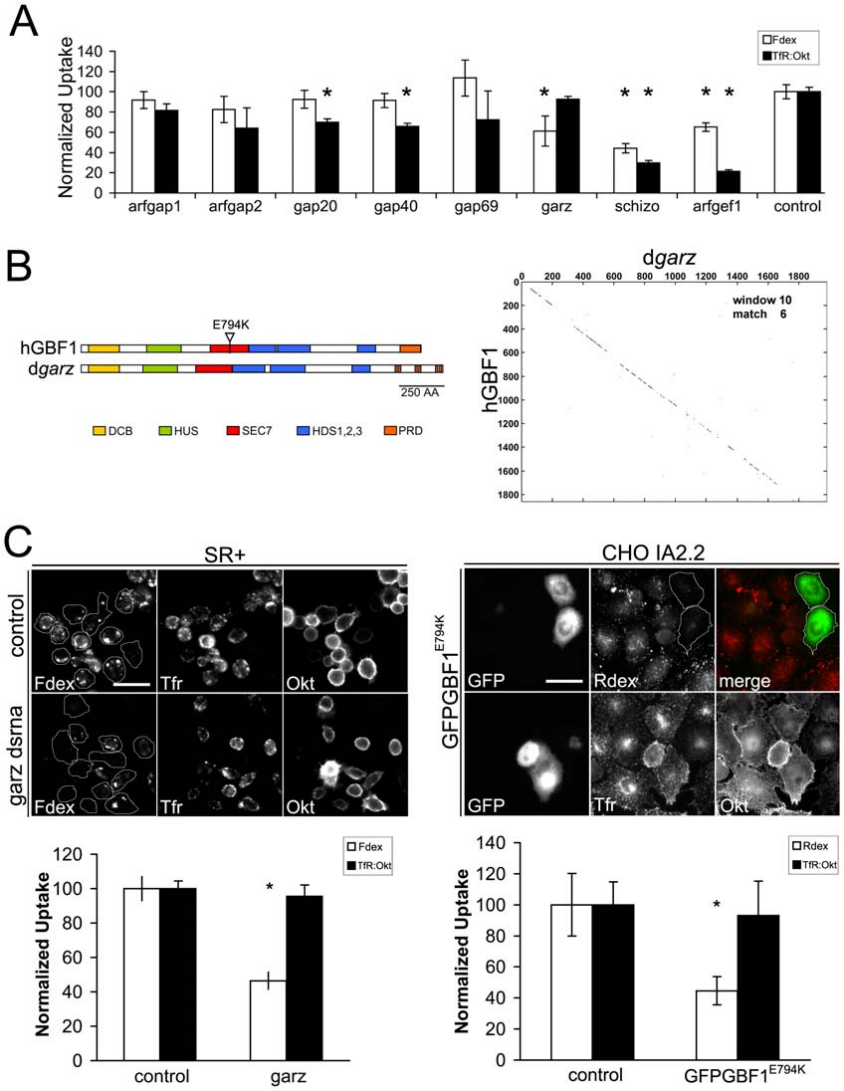
A conserved role for GBF1 in fluid phase uptake in *Drosophila* and mammalian cells. (A) Normalized uptake of Fdex and TfR was measured as before in S2R+ cells treated with dsRNAs corresponding to various predicted Arf GAPs and GEFs from the *Drosophila* genome (corresponding CG numbers are: arfgap1 CG6838; arfgap2 CG16728; gap20 CG4937; gap40 CG8243; gap69 CG4237; garz CG8487; schizo CG32434; arfgef1 CG7578). Notably depletion of the Arf GEF *garz* was singled out because it affected Fdex but not Tf internalization, while other other depletions either had no major effect or affected both pathways significantly. Values are represented as mean±s.e.m. derived from 2 independent experiments with n>200 cells in each condition. Asterix denote P<0.05 by Student's t-test. (B) Cartoon representation of domain structures in human GBF1 (hGBF1)and *Drosophila garz* (d*garz*) proteins. The glutamic acid to lysine substitution mutation within the catalytic Sec7 domain is marked as E794K. A dot plot comparison of hGBF1 and d*garz* primary sequences shows broad conservation throughout the length of the proteins, when a stringent moving window of 6 matches every 10 amino acids is applied. (C) Quantitative uptake of Fdex and TfR in S2R+ cells depleted of *garz* (left panel) or CHO cells overexpressing non-functional GBF1 (GFPGBF1^E794K^ right panel). In the left panel, representative images of S2R+ cells (outlined) treated with control or *garz* dsRNA are shown in 3 channels, depicting uptake/binding with Fdex, TfR and Okt respectively. The bar graph below the panel shows the quantitation of Fdex and TfR uptake normalized to control values. Values are represented as mean±s.e.m. derived from 2 independent experiments with n>200 cells in each condition. Asterix denote P<0.05 by Student's t-test. In the right panel, representative images of CHO cells pulsed with Rdex or TfR and overexpressing GFPGBF1^E794K^ (transfected cells are visible in the GFP channel) are shown. Bars in C = 20 µm.

After ascertaining the presence of an Arf1-mediated pinocytic pathway in *Drosophila* cells and establishing the necessary tools to assay endocytosis, we sought to identify conserved components of this pathway using functional genomic RNAi analysis. To find candidate regulators of Arf1, we screened several orthologous members of the family of Arf GEFs and GAPs in *Drosophila* for defects in quantitative fluid phase and Tf uptake (Figure 7A). While there were several interesting molecules whose depletion affected both Tf and Fdex uptake, we chose to focus on the *Drosophila* Arf GEF *gartenzwerg (garz)* because it was a candidate GTPase regulator which appeared to be exclusively required for Fdex uptake (Figure 7A). Sequence alignment and domain analysis indicates that *Drosophila garz* is the likely ortholog of human GBF1, with over 60% amino acid conservation over the entire length of the protein products (Figure 7B). We used the well-characterized GTPase exchange mutant E794K to perturb GBF1 function [46], [47]. The glutamic acid to lysine substitution within the catalytic Sec7 domain abolishes the nucleotide exchange activity, such that this mutant GEF binds Arf–GDP, but does not catalyze GDP displacement [46]. The overexpression of GBF1^E794K^ in CHO cells caused a specific inhibition of Rdex uptake, whereas internalization of Tf was unaffected (Figure 7C).

### Dynamics of GBF1 reveals a functional association with Arf1

**Figure 8:**
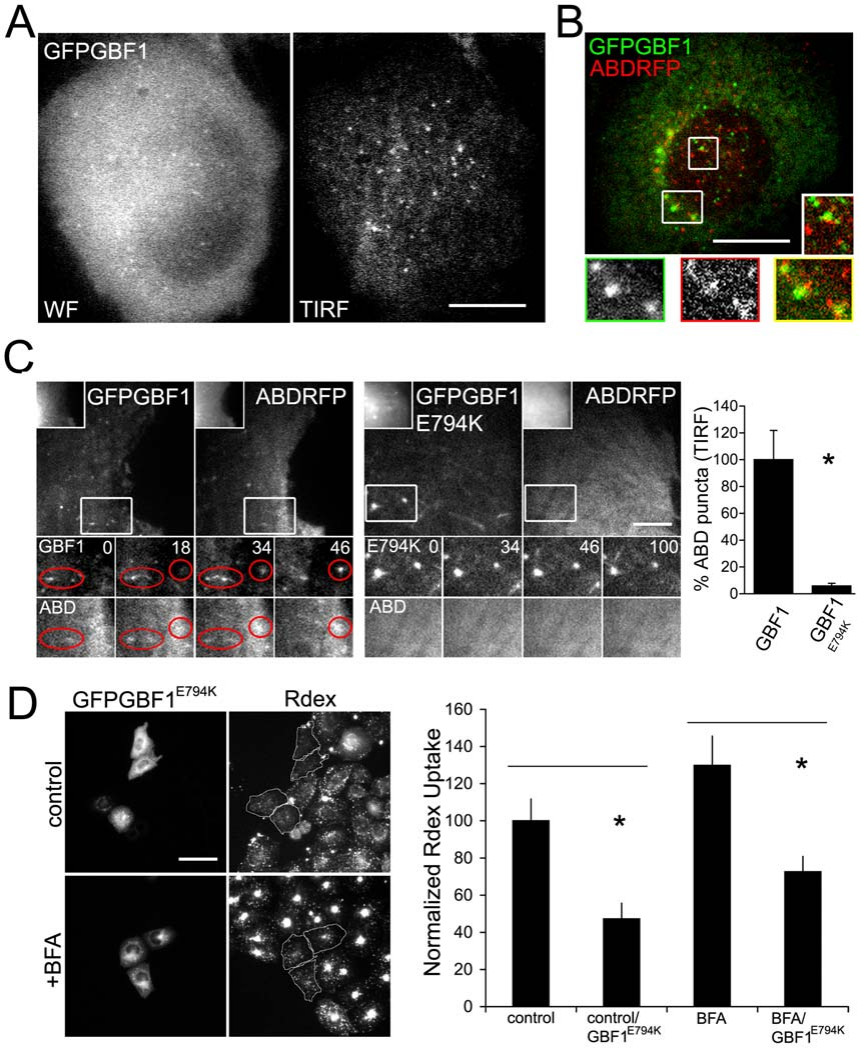
Functional significance of GBF1 dynamics in the vicinity of the plasma membrane. (A) A CHO cell expressing low levels of GFPGBF1 imaged with widefield fluorescence microscopy (WF; left panel) or with TIRF microscopy (right panel) to reveal puncta close to the plasma membrane. (B) Fast sequential two-colour confocal imaging of a live CHO cell expressing GFPGBF1 and ABDRFP (activated Arf sensor; Kumari and Mayor, 2008). The colour merged image is a composite of the most peripheral plane of the cell in each channel (encompassing ∼200 nm in the z-axis, 30 msec between each plane). Two boxed regions in the cell are magnified, the smaller as the merge inset, and the individual channels from the larger region are shown below. (C) Time lapse, sequential TIRF imaging of a CHO cell co-expressing GFPGBF1 and ABDRFP (left panels) or co-expressing GFPGBF1^E794K^ and ABDRFP (right panels). The main reference images in each panel show dual-channel sets of TIRF images for a single time point, with a ∼1 sec gap between channel acquisition. The insets in the reference images show the same plane imaged with widefield microscopy. The boxed regions are magnified and shown as a time series for each channel, with the time elapsed since the first reference frame displayed in seconds. Transient punctate or elongated structures that traverse the evanescent field and acquire both GFPGBF and ABDRFP over the time-lapse are circled in red. In the cell expressing GFPGBF1^E794K^, these structures persist for longer times in the evanescent field (magnified time series; right panel), but do not acquire ABDRFP. Quantitatively, cells expressing GFPGBF1^E794K^ show significantly lower numbers of ABDRFP puncta in the evanescent plane (bar graph in right panel shows mean±s.e.m. values from 20 random planes from movies of 5 cells in each case, expressed as percentage of control).(D) CHO cells transfected with GFPGBF1^E794K^ were treated with Brefeldin A (BFA) or carrier DMSO (control) and assayed for fluid phase (Rdex) uptake. Representative fields with GBF1^E794K^ transfected (outlined in white) and untransfected cells, with or without BFA are shown in the left panel. The bar graph in the right panel shows the corresponding quantitation of fluid phase uptake as normalized to control, untransfected cells (mean±s.e.m.; n = 63,68,60,59; asterix denote P<0.05 by Student's t-test). Bar in A,B = 10 µm, C = 2.5 µm, D = 20 µm. Insets in C are 0.25x, boxed regions are magnified 2x.

GFPGBF1, which reflects the native GBF1 distribution in cells [46], is primarily cytosolic and cycles rapidly on and off membranes during rounds of Arf1 activation at late ER and early Golgi compartments [46], [47]. It follows that the membrane bound fraction of GFP-GBF1 shows extensive colocalization with Arf1 in these organelles [16], [48]. Recently, activated Arf1 has also been detected in punctuate structures close to/at the plasma membrane by TIRF microscopy by using the Arf-binding domain (ABD) of ARHGAP10 fused to GFP as a specific sensor [10]. The specificity of ABD for activated Arf1 stems from the observation that in the evanescent plane, it colocalizes with Arf1 puncta and with nascent pinosomes, and this localization is altered upon overexpression of dominant-negative Arf1 or depletion of Arf1 by shRNA [10]. To examine the role of GBF1 in Arf1 activation near the plasma membrane, cells expressing low levels of GFP-GBF1 were imaged with live TIRF microscopy. A fraction of the total (wide-field) pool of GFP-GBF1 was detectable as punctuate structures in the evanescent field (Figure 8A). In live cells co-expressing ABD-RFP and GFP-GBF1, two colour fast confocal scanning near the plasma membrane revealed a sub-population of puncta which associated with both probes (Figure 8B). In time lapse TIRF microscopy, a fraction of GFP-GBF1 structures that were tracked over time were found to associate with activated Arf1 as masked by ABD-RFP (Figure 8C left panel). Notably, the overexpression of the activation mutant GFP-GBF1^E794K^ caused a striking loss of ABD puncta, leaving only a flat haze in the evanescent field (Figure 8C right panel). This altered distribution of the ABD sensor was similar to that observed previously with overexpression of the activation-impaired Arf1^T31N^ mutant, and of Arf1 shRNA [10]. Qualitatively, the residence time of GFP-GBF1 labeled structures in the evanescent plane was also much longer in the E794K mutant (Figure 8C), which is consistent with previous reports that describe the prolonged membrane association of this cycling-defective protein [46], [47].

GBF1, in keeping with its established role in ER-Golgi transport, could affect endocytosis indirectly by modulating the delivery, to the plasma membrane, of molecules critical to the process. We tested this possibility by first inhibiting Golgi-to-cell surface traffic with Brefeldin A (BFA), and then examining the effect of GBF1 activation on endocytosis. In this assay, wild type cells treated with BFA show increased levels of fluid phase uptake, presumably because factors required for this uptake are released from the Golgi compartment [10]. We found that GFP-GBF1 overexpression alone had no effect on increased fluid uptake following BFA treatment (Figure 8D). By contrast, expression of GFP-GBF1^E794K^ caused a reversal of this BFA-dependent increase (Figure 8D). This reversal, also seen in cells expressing the Arf1^T31N^ mutant [10], implicates BFA-insensitive GBF1 activation in the fluid uptake pathway. Together this data indicates a direct role for GBF1 in Arf1 activation in the vicinity of the plasma membrane.

## Discussion

### Conservation of the GEEC pathway in *Drosophila*

This work demonstrates that the Arf1-mediated pathway in *Drosophila* bears a striking similarity to the GEEC pathway first described in mammalian cells [4], [8], [38]. In both cases, acidic early pinosomes which contain GPI-APs are segregated from transferrin cargo and from Rab5 marked endosomes. The formation of these pinosomes requires optimum levels of cholesterol, actin and a functional Arf1/Cdc42 machinery, and can proceed without functional dynamin and clathrin. Several clathrin-independent pathways other than the Arf1-mediated route have been described in mammalian cells - these depend on caveolin, RhoA, Arf6 or Rac1 [3], [10]. The absence of caveolin in *Drosophila* and the lack of effect on endocytosis upon knockdown of Rho, Arf6 and Rac1 on the specific cargo tested indicate that these molecules may be recruited for specialized pathways that have recently emerged during mammalian evolution. The features of the Arf1-mediated GEEC pathway however, having been conserved across phyla, may represent the hallmarks of a primordial route of entry into metazoan cells.

The present delineation of endocytic pathways in *Drosophila* S2R+ cells, coupled with previous evidence from primary cultures of hemocytes[19] and from S2 cells [39], [49], argues for at least two routes of entry in *Drosophila* cells: a Tf and mBSA carrying pathway that requires clathrin and dynamin; and a bulk fluid phase uptake pathway that carries a large fraction of GFP-GPI. At present, we cannot rule out the presence of additional endocytic pathways, since a fraction of GFP-GPI is detected as separate from fluid cargo, and some Tf continues to enter cells in the absence of dynamin. The use of additional probes in combination with specific gene knockdowns will help to dissect these findings further. Following internalization, both receptor and fluid pathways converge onto Rab5 containing endosomes, where heterotypic and homotypic mixing occurs. The kinetics of mixing of transferrin/mBSA and fluid phase cargo in S2R+ cells are similar to those described for *Drosophila* hemocytes, where mixed cargo reached late endosomes within 5 min [19]. Compared to CHO cells, TfR recycling occurs at faster rates in S2R+ cells. While we have assumed, based on a parsimonious fit to the data that TfR efflux occurs as a first order process, a two component model that allows for fast and slow recycling steps can also be proposed [50]. A fast recycling step is consistent with the kinetics of Rab5 acquisition, which peaks at 2 min post-internalization and with the observed regurgitation of ∼40% of internalized bulk fluid phase over 5 min. In this aspect, S2R+ cells, which are related to the macrophage S2 isolates [26], appear similar to specialized phagocytic cell types where fast receptor recycling is common [51], [52]. While similarities exist between phagocytosis and macropinocytosis in mammalian cells [53] and molecules such as actin and Cdc42 are common to macropinocytosis and Arf1-mediated pinocytosis, the fluid phase pathway described here in *Drosophila* appears to be unstimulated and does not depend on Rac1 activity. We suggest that this pathway, akin to the GEEC pathway described in mammalian cells, is a distinct constitutive pinocytic process [4].

Our observations of differential, time-dependent sensitivity of fluid uptake to Latrunculin versus Jasplakinolide, as well as dependence of the process on Cdc42, suggest the requirement for a highly dynamic population of actin for mediating pinocytic entry. Recent evidence indicates that cortical actin dynamics drives the complexation of GPI-anchored proteins (GPI-APs) on the cell surface in a cholesterol dependent manner [9], and Cdc42 activity as well as the proper organization of GPI-APs is a requirement for internalization via the GEEC pathway [7], [8]. Our description of a cholesterol-auxotrophic cell line defective in fluid phase uptake should allow further investigation of the relationship between cholesterol, GPI-AP organization and Cdc42 activity.

### Role of GBF1 in Arf1-mediated endocytosis

There are several lines of evidence for the involvement of the GTPase exchange factor GBF1 in Arf1-mediated endocytosis. The depletion of the GBF1 ortholog *garz* in S2R+ cells results in specific inhibition of Arf1-dependent fluid uptake. Concurrently, expression of the exchange defective GBF1^E794K^ mutant results in the same defect in CHO cells. A fraction of GBF1 colocalizes with ABD-RFP - a specific sensor for activated Arf1- in the evanescent plane (∼100 nm proximity to the plasma membrane). Notably, ABD puncta are lost from the evanescent plane upon expression of GBF1^E794K^, thus providing a direct link between GBF1 exchange activity, Arf1 activation at/close to the plasma membrane, and Arf1-mediated endocytosis.

GBF1 is known to cycle rapidly between membranes and cytosol (t_1/2_∼17 s; [46]). Membrane recruitment occurs via an unknown receptor(s) and is independent of Arf-GDP recruitment, while membrane dissociation occurs shortly after GTP exchange [46], [48]. Sustained activation of Arf1 would require multiple rounds of GBF1 cycling and GTP exchange, as GBF1 cycles on/off membranes faster than Arf1 [46]. As such, the fraction of GBF1 associated with activated Arf1 at any point in space and time would be relatively small. We were able to detect this small fraction in the evanescent field with the aid of sensitive cameras and low expression levels of GBF1 and ABD. Since the majority of membrane-associated GBF1 and ABD was located in pericentriolar Golgi and transitional ER compartments (which rarely traverse the evanescent plane in TIRF imaging), good contrast was achievable near the plasma membrane where the concentration of GBF1 and ABD was relatively low.

Since GBF1 was originally discovered as a GEF required for ER-Golgi transport in the secretory pathway [54], its role in endocytosis as ascribed here is novel. Extending an earlier finding that Arf1 activation continues to be required for endocytosis even after disruption of the early secretory pathway with BFA [10], we have shown here that the GEF activity of GBF1 is an additional requirement for this to occur. However, this raises the paradoxical issue of how GBF1 can still activate Arf1 in the presence of BFA, which is a non-competitive inhibitor that stabilizes a Arf-GDP-BFA-GEF complex, and causes morphological defects *in vivo* that mimic the loss of Arf1-GTP ([48] and refs. therein). As multiple GEFs can activate Arf1, and several are targeted by BFA [13], the routinely used concentrations of this drug may be sufficient to inhibit ER-Golgi traffic and disrupt Golgi complex morphology, but may not inhibit all GBF1 activity *in vivo*. Equally, it is possible that GBF1 sensitivity to BFA is modulated by its membrane receptors, and the membrane recruitment of GBF1 at the ER-Golgi route is more sensitive to BFA than membrane recruitment at the plasma membrane. A third non-mutually exclusive possibility is that the presence of excess Arf1 that has been released from the ER-Golgi pool upon BFA addition stimulates GBF1 activity at the plasma membrane, which compensates for GBF inhibition. Identification and characterization of the receptor(s) and the membrane recruitment mechanism for GBF1 would help to distinguish between these possibilities.

A role for GBF1 in endocytosis is not without precedent. In *Arabidopsis*, the GBF1-ortholog GNL1 has a conserved role at the Golgi but is also required for BFA-insensitive endocytosis at the plasma membrane [55]. Moreover, the *Drosophila* GBF1 ortholog *garz* has been identified as a candidate in genomic screens which tested the entry and infection of the pathogens *Listeria monocytogenes* [56] and *Candida albicans* [57], [58]. While direct involvement of candidate genes in pathogen entry was not tested in these screens, our finding that *garz* affects Arf1-mediated endocytosis in *Drosophila* implicate this pathway as a mode of entry for these pathogens. There is growing evidence that Arf1, GBF1 and part of the COPI machinery can be recruited not only onto transitional ER and the Golgi, but also onto other membranes where they can modulate trafficking or modify membranes (replication complexes during picornaviral infection - [59], [60]; lipid bodies – [61], [62]. While both replication complexes and lipid bodies are probably ER-derived membranes [63], [64], our results suggest that Arf1 and GBF1 may also act together during endocytic events at the plasma membrane. In this regard, it would be interesting to test the involvement of components of the COPI machinery in Arf1-mediated endocytosis.

### Conclusion

The availability of heterologous gene expression and functional genomic RNAi in the S2R+ line, combined with high-resolution imaging promises to be a powerful approach to study endocytosis in *Drosophila*. These methods have allowed us to systematically identify and define the role of a novel molecule in GEEC endocytosis. The demonstration of conserved pathways in *Drosophila* indicates that these findings can be broadly applicable.

## Supporting Information

Figure S1 Automated quantitation of uptake assay measurements. (A) High penetrance of a dsRNA induced phenotype in S2R+ cells cultured on dishes. Images taken from a single field of S2R+ cells treated with control dsRNA or dsRNA against pavarotti, which encodes a kinesin like protein required for cytokinesis[1]. Note the accumulation of abnormally large multinucleate cells with pavarotti dsRNA as shown in the brightfield/Hoechst-stained nuclear channels. (B) Stepwise procedure for performing automated cell identification using the MATLAB image processing toolbox. The nuclear image (Hoechst) is thresholded using Otsu's method [2] and an empirically-determined correction factor to create a binary mask (nuclear binary mask). This binary mask is subjected to a Euclidean distance transform followed by a watershed transform, generating the first crude stage of cell segmentation (nuclear distance transform, ndt). A Sobel edge-detection filter is applied to the Okt9 surface label image (Okt9) to enhance cell boundaries. The watershed and the nuclear mask are superimposed upon the enhanced Okt9 image as local minima (local minima of ndt+Okt9). A final watershed transform is applied to this combined image to generate cell outlines which are then filled in (surface mask). (C) Scatterplot comparing the performance of automated intensity/cell quantitation vs manual quantitation. 24 dishes were pulsed with Fdex for different times to generate a ∼10fold range of fluorescence intensity values across cells. Each point represents the mean±s.e.m. intensity/cell in a dish (with measurements from >100 cells from each dish) calculated manually or via MATLAB routines. The two methods show linear correlation over a wide range of cell intensities. Bar in A = 20 µm, B = 5 µm. References: 1.Adams, R.R., et al., pavarotti encodes a kinesin-like protein required to organize the central spindle and contractile ring for cytokinesis. Genes Dev, 1998. 12(10): p. 1483-94. 2. Otsu, N., A Threshold Selection Method from Gray-Level Histograms. IEEE Transactions on Systems, Man, and Cybernetics 1979. 9: p. 62–66.
(3.45 MB TIF)

Figure S2 Controls for trafficking assays. (A) Direct comparison of the mixing of Fdex and TfR probes at various pulse and chase times in S2R+ cells expressing TfR alone or S2R+ cells expressing TfR and Rab5 GFP. Fdex was co-pulsed with A568Tf (TfR) in wild-type S2R+ cells or S2R+ cells expressing Rab5 GFP for 30 s and 60 s, or pulsed for 60 s and chased for 1 min or 4 min in complete medium. The histogram shows quantified colocalization indices of: the fraction of Fdex-labeled endosomes that co-localized with Tf. Bars represent mean±s.e.m pooled from 2 independent experiments with >20 cells each. (B) Approach to steady state assay of TfR in cells treated with cyclohexamide. S2R+ cells expressing TfR were pretreated with 75 uM cyclohexamide (Chx) for 2 hrs to block protein synthesis. They were then pulsed with A647Tf for different times according to the ‘approach to steady state’ assay (see Methods and Figure 2A) in the presence of Chx. Data for control S2R+ cells expressing TfR, but without Chx pretreatment was taken from Figure 3A and is plotted for comparison. Each data point represents the mean±s.e.m. of a replicate experiment from at least 50 cells/replicate. Points have been fit to y = 0.381+0.666(1 - e^-0.216t^), R^2^ = 0.922 for TfR and y = 0.332+0.698(1 - e^-0.18t^), R^2^ = 0.994 for Tfr with Chx.
(0.62 MB TIF)
