# Supplementary figures and images for: Analysis of Endocytic Pathways in Drosophila Cells Reveals a Conserved Role for GBF1 in Internalization via GEECs
Source: PLoS One. 2009 Aug 26;4(8):e6768. doi: 10.1371/journal.pone.0006768 (PMC2728541; doi:10.1371/journal.pone.0006768)

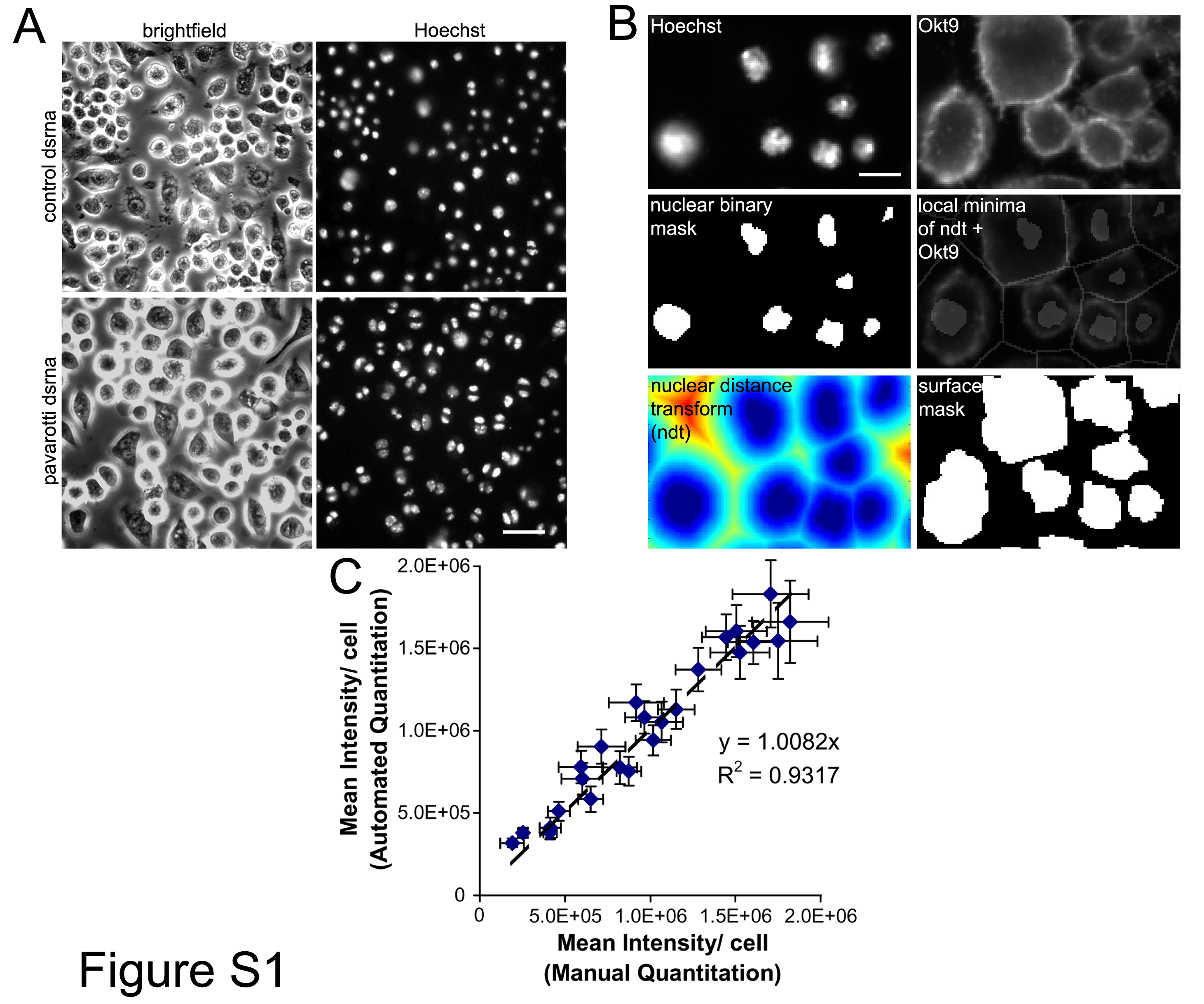

Supplement: Figure S1 — Automated quantitation of uptake assay measurements. (A) High penetrance of a dsRNA induced phenotype in S2R+ cells cultured on dishes. Images taken from a single field of S2R+ cells treated with control dsRNA or dsRNA against pavarotti, which encodes a kinesin like protein required for cytokinesis[1]. Note the accumulation of abnormally large multinucleate cells with pavarotti dsRNA as shown in the brightfield/Hoechst-stained nuclear channels. (B) Stepwise procedure for performing automated cell identification using the MATLAB image processing toolbox. The nuclear image (Hoechst) is thresholded using Otsu's method [2] and an empirically-determined correction factor to create a binary mask (nuclear binary mask). This binary mask is subjected to a Euclidean distance transform followed by a watershed transform, generating the first crude stage of cell segmentation (nuclear distance transform, ndt). A Sobel edge-detection filter is applied to the Okt9 surface label image (Okt9) to enhance cell boundaries. The watershed and the nuclear mask are superimposed upon the enhanced Okt9 image as local minima (local minima of ndt+Okt9). A final watershed transform is applied to this combined image to generate cell outlines which are then filled in (surface mask). (C) Scatterplot comparing the performance of automated intensity/cell quantitation vs manual quantitation. 24 dishes were pulsed with Fdex for different times to generate a ∼10fold range of fluorescence intensity values across cells. Each point represents the mean±s.e.m. intensity/cell in a dish (with measurements from >100 cells from each dish) calculated manually or via MATLAB routines. The two methods show linear correlation over a wide range of cell intensities. Bar in A = 20 µm, B = 5 µm. References: 1.Adams, R.R., et al., pavarotti encodes a kinesin-like protein required to organize the central spindle and contractile ring for cytokinesis. Genes Dev, 1998. 12(10): p. 1483-94. 2. Otsu, N., A Threshold Selection Me [file pone.0006768.s001.tif]

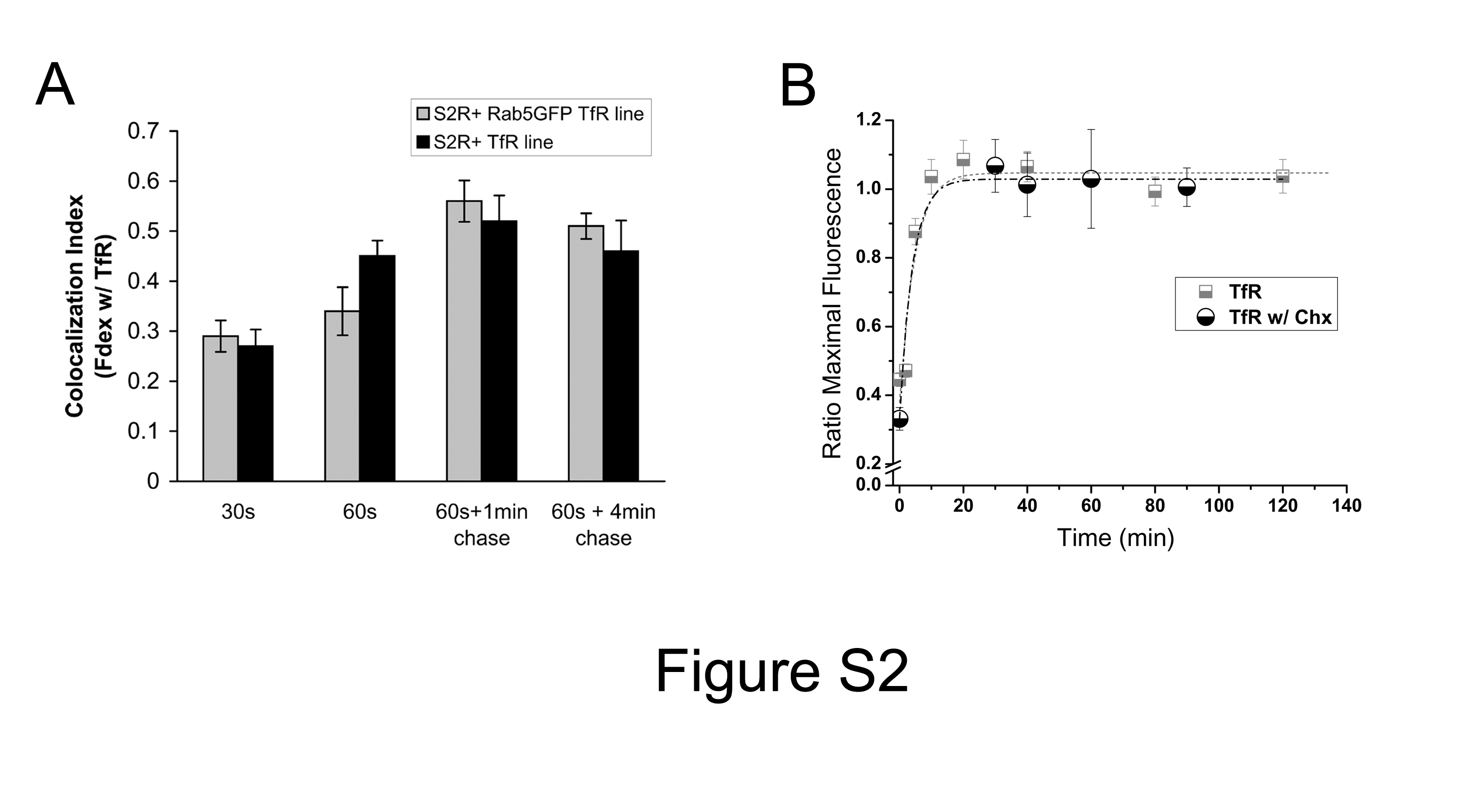

Supplement: Figure S2 — Controls for trafficking assays. (A) Direct comparison of the mixing of Fdex and TfR probes at various pulse and chase times in S2R+ cells expressing TfR alone or S2R+ cells expressing TfR and Rab5 GFP. Fdex was co-pulsed with A568Tf (TfR) in wild-type S2R+ cells or S2R+ cells expressing Rab5 GFP for 30 s and 60 s, or pulsed for 60 s and chased for 1 min or 4 min in complete medium. The histogram shows quantified colocalization indices of: the fraction of Fdex-labeled endosomes that co-localized with Tf. Bars represent mean±s.e.m pooled from 2 independent experiments with >20 cells each. (B) Approach to steady state assay of TfR in cells treated with cyclohexamide. S2R+ cells expressing TfR were pretreated with 75 uM cyclohexamide (Chx) for 2 hrs to block protein synthesis. They were then pulsed with A647Tf for different times according to the ‘approach to steady state’ assay (see Methods and Figure 2A) in the presence of Chx. Data for control S2R+ cells expressing TfR, but without Chx pretreatment was taken from Figure 3A and is plotted for comparison. Each data point represents the mean±s.e.m. of a replicate experiment from at least 50 cells/replicate. Points have been fit to y = 0.381+0.666(1 - e-0.216t), R2 = 0.922 for TfR and y = 0.332+0.698(1 - e-0.18t), R2 = 0.994 for Tfr with Chx. (0.62 MB TIF) [file pone.0006768.s002.tif]
